# Significant association between decreased ALDH2 activity and increased sensitivity to genotoxic effects in workers occupationally exposed to styrene

**DOI:** 10.18632/oncotarget.9502

**Published:** 2016-05-20

**Authors:** Zuquan Weng, Megumi Suda, Mei Wan, Xing Zhang, Dongzhu Guan, Peiqing Zhao, Yuxin Zheng, Muneyuki Miyagawa, Rui-Sheng Wang

**Affiliations:** ^1^ College of Biological Science and Engineering, Fuzhou University, Fuzhou, China; ^2^ Japan National Institute of Occupational Safety and Health, Kawasaki, Japan; ^3^ National Institute of Occupational Health and Poison Control, China CDC, Beijing, China; ^4^ Food and Drug Administration of Beijing Fengtai District, Beijing, China; ^5^ Beijing Guoji-Zhongyi Hospital, Beijing, China; ^6^ Present address: Department of Sport and Medical Science, Faculty of Medical Technology, Teikyo University, Tokyo, Japan

**Keywords:** styrene, ALDH2 polymorphisms, aldehyde metabolism, genotoxicity, cancer risk

## Abstract

ALDH2 is involved in the metabolism of styrene, a widely used industrial material, but no data are available regarding the influence of this enzyme on the metabolic fate as well as toxic effects of this chemical. In this study, we recruited 329 workers occupationally exposed to styrene and 152 unexposed controls. DNA strand breaks, DNA-base oxidation in leukocytes and urinary 8-hydroxydeoxyguanosine (8-OH-dG) were assayed as biomarkers to measure genotoxic effects. Meanwhile, we examined the genetic polymorphisms, including *ALDH2, EXPH1, GSTM1, GSTT1* and *CYP2E1*, and also analyzed the levels of styrene exposure through detecting urinary styrene metabolites and styrene concentration in air. In terms of DNA damage, the three genotoxic biomarkers were significantly increased in exposed workers as compared with controls. And the styrene-exposed workers with inactive ALDH2 *2 allele were subjected to genotoxicity in a higher degree than those with ALDH2 *1/*1 genotype. Also, lower levels of urinary styrene metabolites (MA + PGA) were observed in styrene-exposed workers carrying ALDH2 *2 allele, suggesting slower metabolism of styrene. The polymorphisms of other enzymes showed less effect. These results suggested that styrene metabolism and styrene-induced genotoxicity could be particularly modified by *ALDH2* polymorphisms. The important role of *ALDH2* indicated that the accumulation of styrene glycoaldehyde, a possible genotoxic intermediate of styrene, could account for the genotoxicity observed, and should be taken as an increased risk of cancer.

## INTRODUCTION

Styrene is a widely used material in production of plastic, rubber, fiberglass and etc. Occupational populations may be exposed to higher concentration of styrene, especially in the reinforced plastic industry [1, 2]. On the other hand, public exposure to this chemical may also occur due to mainstream cigarette smoke, engine exhausts, carpets and food packaging [3–6]. Inhalation is a major route for both occupational and environmental exposure to styrene [7].

Over the decades, many efforts have been made to study styrene-induced adverse health effects. In some review articles [6, 8, 9], convincing evidences have been shown that styrene induces lung cancer in mice, while evidence in humans is unclear. Many studies, but not all, have shown that styrene is genotoxic. International Agency for Research on Cancer (IARC) has classified styrene in group 2B as a possible human carcinogen, and its metabolite, styrene-7,8-oxide (SO), has been classified in group 2A, probably carcinogenic to humans [10].

Genetic variation appeared to be an important reason for the uncertainty of styrene-induced toxicity. So far the polymorphisms of some enzymes involved in styrene metabolism, such as P-450 cytochromes, microsomal epoxide hydrolase (EXPH), and glutathione S-transferases (GSTs), have been studied and reviewed for the impacts on genotoxic risk of styrene-exposed populations [9]. In general, these polymorphisms might modify individual susceptibility to styrene-induced DNA damage. But it was difficult to draw any conclusion on possible associations between genes polymorphisms and biomarkers of styrene-induced DNA damage, as most studies evaluated complex gene-gene and gene-environment interactions with small samples.

**Figure 1:**
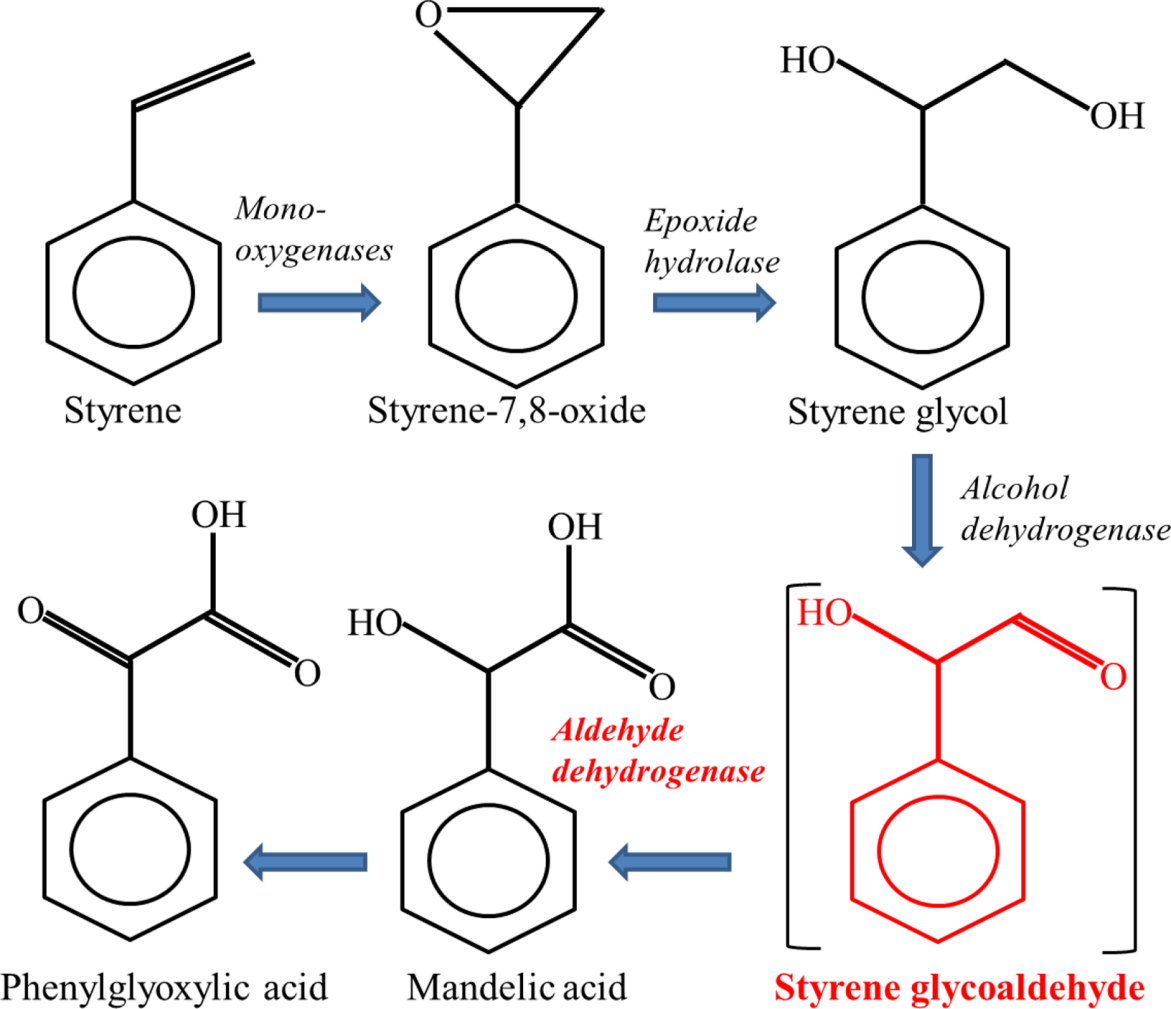
Major pathway of styrene metabolism

It is well-known that human aldehyde dehydrogenases (ALDHs) are involved in the metabolic pathways of endogenous and exogenous compounds. In addition, ALDH activity has been reported to represent a functional marker for cervical cancer stem cells as well as a target for novel cervical cancer therapies [11]. In the process of styrene metabolism, ALDHs participate in the formation of mandelic acid (MA) and phenylglyoxylic acid (PGA) (Figure 1), which account for more than 95% of the urinary metabolites of styrene [9]. So far, however, no data are available regarding the effects of *ALDHs* polymorphisms on metabolism and toxicity of styrene. In particular, deficient ALDH2 activity caused by mutant allele (ALDH2 *2) [12] may dramatically change the metabolism of styrene and its intermediates, which may in turn lead to different toxic effects among individuals exposed to styrene. The frequencies of *ALDH2* genotypes vary in different ethnical groups, and the variant allele ALDH2 *2 is highly prevalent (about 30–50%) in East Asian populations, while almost all Caucasians carried the functional ALDH2 *1/*1 genotype [13–15]. Previous studies have indicated that ALDH2 *2 allele increased the risk for alcohol-related cancers [12, 16]. Moreover, the ethnical difference in *ALDH2* polymorphisms could significantly influence the levels of biological exposure indices of organic solvent in urinary and blood, such as toluene, perchloroethylene and methyl ethyl ketone [17–19]. As a result, Orientals tend to have lower concentrations of urinary PGA than Caucasian under inhalational exposure to styrene [20]. Taken together, the valuable information above prompted us to examine whether *ALDH2* polymorphisms exert any modifying effect on the metabolic processes and genotoxic effects of styrene.

In this study, we recruited a relative large sample of 329 workers occupationally exposed to styrene and 152 unexposed controls. A combination of three genotoxic biomarkers, DNA strand breaks, DNA-base oxidation in leukocytes, and urinary 8-OH-dG, were examined for accurate assessment of DNA damage related to styrene. Urinary MA+PGA concentrations and styrene concentration in air were used to characterize styrene exposure level.

## RESULTS

### Study subjects and styrene exposure

The exposed subjects were divided into three groups by workplaces. No significant differences were found between controls and exposed subjects in age, gender, smoking or drinking rate (Table 1). The mean styrene concentrations in air at workplace A, B and C were 26.21 ± 14.18, 36.75 ± 19.27 and 57.17 ± 31.01 ppm, respectively. And the total mean concentration was 40.04 ± 25.43 ppm. Meanwhile, the highest mean value of urinary MA+PGA (119.64 ± 127.18 mg/g creatinine) was found in workplace C, and the total mean value in three workplaces was 91.56 ± 146.55 mg/g creatinine.

**Table 1:** Characteristics of the study subjects and characterization of styrene exposure

		Control	Workplace A	Workplace B	Workplace C	All exposed
No. of subjects		152	120	115	94	329
Age	Mean ± SD (years)	38.43 ± 11.80	36.58 ± 8.55	38.74 ± 8.25	39.86 ± 7.42	38.27 ± 8.22
Gender	Male No. (%)	91 (59.87)	61 (50.83)	83 (72.17)	68 (72.34)	212 (64.44)
	Female No. (%)	61 (40.13)	59 (49.17)	32 (27.83)	26 (27.66)	117 (35.56)
Smoking status	Non-smokers No. (%)	118 (77.63)	110 (91.67)	100 (86.96)	70 (74.49)	280 (85.11)
	Smokers No. (%)	34 (22.37)	10 (8.33)	15 (13.04)	24 (25.51)	49 (14.89)
Drinking status	Non-drinkers No. (%)	119 (78.29)	109 (90.83)	101 (87.83)	75 (79.78)	285 (86.63)
	Drinkers No. (%)	33 (21.71)	11 (9.17)	14 (12.17)	19 (20.21)	44 (13.37)
Years of employment (Mean ± SD)		-	6.96 ± 6.78	7.28 ± 8.05	8.91 ± 8.08	7.63 ± 7.64
Styrene at workplace (Mean ± SD; ppm)		n.d	26.21 ± 14.18	36.75 ± 19.27	57.17 ± 31.01	40.04 ± 25.43
MA+PGA (Mean ± SD; mg/g creatinine)		n.d	72.44 ± 153.62	87.88 ± 151.61	119.64 ± 127.18	91.56 ± 146.55

Note: MA+ PGA: mandelic acid + phenylglyoxylic acid; n.d.: not detectable.

### Biomarkers for DNA damage related to styrene exposure

The levels of DNA strand breaks, DNA- base oxidation in leukocytes, and urinary 8-OH-dG concentrations were presented in Table 2. The mean level of DNA strand breaks in leukocytes, measured by a standard comet assay, was significantly higher in exposed workers (workplace A: 12.85 ± 3.75, workplace B: 13.02 ± 3.18, workplace C: 13.99 ± 2.92, and all exposed: 13.24 ± 3.36) than in controls (11.71 ± 3.88). As well, using Fpg-modified comet assay, we detected higher DNA-base oxidation in leukocytes among exposure groups as compared to control group. Furthermore, urinary 8-OH-dG, representing the whole body oxidative DNA damage, also showed significantly higher levels in various exposed groups (workplace A: 4.51 ± 1.61, workplace B: 4.72 ± 2.01, workplace C: 5.96 ± 2.08, and all exposed: 5.00 ± 1.99) than in controls (3.95 ± 1.42). These results collectively suggested the positive correlation between genetic damage and exposure level of styrene.

**Table 2:** DNA damage of leukocytes and biomarker of urinary nuclei acid oxidation in controls and styrene-exposed workers

	Study subjects													
	Controls		Styrene-exposed workers											
			Workplace A			Workplace B			Workplace C			All exposed		
	No.	Mean ± SD	No.	Mean ± SD	*P*-value	No.	Mean ± SD	*P*-value	No.	Mean ± SD	*P*-value	No.	Mean ± SD	*P*-value
Tail Intensity	145	11.71 ± 3.88	119	12.85 ± 3.75	1.63E-02*	114	13.02 ± 3.18	2.96E-03**	94	13.99 ± 2.92	5.02E-07***	327	13.24 ± 3.36	5.30E-05***
Net Fpg DNA damage	145	6.08 ± 1.92	119	6.71 ± 2.18	1.40E-02*	114	6.85 ± 2.35	5.02E-03**	94	7.55 ± 2.14	8.12E-08***	327	7.00 ± 2.25	7.81E-06***
Urinary 8-OH-dG(ng/mg creatinine)	148	3.95 ± 1.42	114	4.51 ± 1.61	3.11E-03**	112	4.72 ± 2.01	3.69E-04***	91	5.96 ± 2.08	1.87E-13***	317	5.00 ± 1.99	2.43E-10***

* *p* < 0.05,

** *p* < 0.01,

*** *p* < 0.001, based on independent samples *t* - test, and compared with the corresponding controls.

### Association between *ALDH2* polymorphisms and the effect of styrene exposure

The distribution of genotypes of *ALDH2* and other enzyme genes was summarized in Table S1. As shown in Table 3, compared to wild-type genotype ALDH2 *1/*1, a significant lower mean value of PGA+MA excretion was found in the exposed individuals (all workplaces combined) carrying the variant allele ALDH2 *2. In addition, this significant difference could still be found in respective workplaces representing relatively small size of samples.

**Table 3:** Effects of *ALDH2* polymorphisms on urinary excretion of styrene specific metabolites and the levels of various parameters of genetic damage in control and styrene-exposed workers

Group	ALDH2 genotypes	Urinary MA+PGA (mg/g creatinine)			Urinary 8-OH-dG (ng/mg creatinine)			Tail Intensity			Net Fpg DNA damage		
		No.	Mean ± SD	*P*-value	No.	Mean ± SD	*P*-value	No.	Mean ± SD	*P*-value	No.	Mean ± SD	*P*-value
Control	ALDH2 1*/1*		n.d		94	3.88 ± 1.41	-	96	11.42 ± 4.05	-	96	5.91 ± 1.98	-
	ALDH2 1*/2*		n.d		42	3.91 ± 1.47	8.88E-01	43	12.04 ± 3.57	3.71E-01	43	6.38 ± 1.88	1.88E-01
	ALDH2 2*/2*		n.d		5	3.74 ± 0.77	7.30E-01	6	13.93 ± 2.81	8.30E-02	6	6.68 ± 0.91	1.05E-01
	ALDH2 1*/2*+2*/2*		n.d		47	3.89 ± 1.41	9.38E-01	49	12.27 ± 3.52	1.96E-01	49	6.41 ± 1.79	1.25E-01
Workplace A	ALDH2 1*/1*	78	86.94 ± 181.02	-	78	4.50 ± 1.69	-	83	12.32 ± 3.75	-	83	6.63 ± 2.26	-
	ALDH2 1*/2*	30	41.56 ± 46.57	4.35E-02*	30	4.38 ± 1.53	7.24E-01	31	13.87 ± 3.65	4.92E-02*	31	6.85 ± 2.09	6.18E-01
	ALDH2 2*/2*	4	36.35 ± 32.46	6.85E-02	5	5.13 ± 0.88	1.95E-01	5	15.39 ± 2.26	3.44E-02*	5	7.11 ± 1.60	5.51E-01
	ALDH2 1*/2*+2*/2*	34	40.94 ± 44.77	3.82E-02*	35	4.49 ± 1.47	9.69E-01	36	14.08 ± 3.50	1.58E-02*	36	6.89 ± 2.01	5.32E-01
Workplace B	ALDH2 1*/1*	83	99.60 ± 161.81	-	80	4.36 ± 1.47	-	83	12.58 ± 3.05	-	83	6.52 ± 2.28	-
	ALDH2 1*/2*	29	60.22 ± 123.74	1.80E-01	29	5.63 ± 2.94	3.30E-02*	29	14.16 ± 3.36	3.06E-02*	29	7.61 ± 2.36	3.58E-02*
	ALDH2 2*/2*	2	15.29 ± 2.84	9.57E-06***	2	5.46 ± 0.04	4.71E-09***	2	15.14 ± 1.43	2.11E-01	2	9.55 ± 1.62	2.16E-01
	ALDH2 1*/2*+2*/2*	31	57.32 ± 120.07	1.35E-01	31	5.62 ± 2.84	2.45E-02*	31	14.22 ± 3.27	1.85E-02*	31	7.74 ± 2.35	1.64E-02*
Workplace C	ALDH2 1*/1*	63	135.35 ± 144.31	-	63	5.55 ± 1.87	-	63	13.10 ± 2.63	-	63	7.10 ± 2.06	-
	ALDH2 1*/2*	27	88.69 ± 70.02	4.22E-02*	26	6.83 ± 2.32	1.64E-02*	29	15.62 ± 2.66	8.82E-05***	29	8.36 ± 2.05	8.06E-03**
	ALDH2 2*/2*	2	42.85 ± 3.30	4.09E-06***	2	7.63 ± 1.36	2.60E-01	2	18.17 ± 1.50	1.05E-01	2	10.16 ± 0.98	1.06E-01
	ALDH2 1*/2*+2*/2*	29	85.53 ± 68.50	2.72E-02*	28	6.89 ± 2.26	8.52E-03**	31	15.79 ± 2.66	2.14E-05***	31	8.48 ± 2.04	3.10E-03**
All exposed	ALDH2 1*/1*	224	105.25 ± 164.67	-	221	4.75 ± 1.74	-	229	12.63 ± 3.22	-	229	6.72 ± 2.21	-
	ALDH2 1*/2*	86	62.65 ± 87.51	3.57E-03**	85	5.55 ± 2.51	7.33E-03**	89	14.54 ± 3.31	7.07E-06***	89	7.59 ± 2.23	2.00E-03**
	ALDH2 2*/2*	8	32.71 ± 24.04	4.20E-06***	9	5.76 ± 1.33	5.31E-02*	9	15.95 ± 2.17	1.50E-03**	9	8.33 ± 1.97	4.04E-02*
	ALDH2 1*/2*+2*/2*	94	60.10 ± 84.34	1.43E-03**	94	5.57 ± 2.42	3.14E-03**	98	14.67 ± 3.24	4.86E-07***	98	7.66 ± 2.21	5.28E-04***

* *p* < 0.05,

** *p* < 0.01,

*** *p* < 0.001, based on independent samples *t* - test, and compared with the corresponding wild-type genotype. Data are expressed as mean ± S.E. n.d.: not detectable.

As for styrene-induced DNA damage, exposed workers with ALDH2 *2 allele had significantly higher levels of DNA strand breaks, DNA-base oxidation in leukocytes and urinary 8-OH-dG than individuals of wild-type genotype. When checking each workplace, significant effects of ALDH2 *2 allele were found in workplace B and C, representing middle and high level exposure. On the other hand, in workplace A with low level exposure, only DNA-base oxidation level was significantly high in ALDH2 *2 allele carriers, but not DNA strand breaks and urinary 8-OH-dG. These results were further confirmed by multivariate linear regression models including sex, age, years of employment, smoking and alcohol drinking (Table 4). In contrast, *ALDH2* polymorphisms did not influence DNA damage in controls.

**Table 4:** Multivariate linear regression models evaluating the relationship between *ALDH2* genotype and the levels of styrene specific metabolites and various parameters of genetic damage

ALDH2 genotypes	Urinary MA+PGA (mg/g creatinine))			Urinary 8-OH-dG (ng/mg creatinine)			Tail Intensity			Net Fpg DNA damage		
	No	Coefficient	*P*-value	No	Coefficient	*P*-value	No	Coefficient	*P*-value	No	Coefficient	*P*-value
ALDH2 *1/*1	221	−39.37	1.33E-02	221	0.72	7.55E-04	229	1.83	2.01E-07	229	0.85	3.44E-04
ALDH2 *1/*2	86			85			89			89		
ALDH2 *2/*2	8			9			9			9		
ALDH2 *1/*1	221	−43.85	1.51E-02	221	0.83	6.61E-04	229	2.04	4.50E-07	229	0.95	4.67E-04
ALDH2 *1/*2 + *2/*2	94			94			98			98		

The linear model was adjusted for sex, age, years of employment, smoking and alcohol drinking, thus determining the regression coefficient and significance level (*p < 0.05, **p < 0.01, ***p < 0.001). The samples with missing values regarding any variable in the linear model were excluded.

### Association between other genetic polymorphisms and biomarkers of exposure and genotoxic effect

Table 5 showed that none of three biomarkers representing genotoxic endpoints was significantly affected by *CYP2E1*, *GSTM1*, *GSTT1* or *EPHX1* polymorphisms in all styrene-exposed workers. Only the variant homozygous *CYP2E1* Rsal c2/c2 and Dral C/C were found to be associated with lower concentration of urinary MA+PGA. We also examined these genetic polymorphisms for each workplace as well as controls (Tables S2–S5). Significant signals were observed only in workplace B, but not in workplace A, C and controls.

**Table 5:** Effects of *GSTM1, GSTT1, EPHX1* and *CYP2E1* polymorphisms on urinary excretion of styrene specific metabolites and the levels of various parameters of genetic damage in all styrene-exposed workers

All Exposed	Urinary MA+PGA (mg/g creatinine)			Urinary 8-OH-dG (ng/mg creatinine)			Tail Intensity			Net Fpg DNA damage		
	No.	Mean ± SD	*P*-value	No.	Mean ± SD	*P*-value	No.	Mean ± SD	*P*-value	No.	Mean ± SD	*P*-value
*GSTM1* (plus)	127	108.45 ± 149.71	-	126	5.01 ± 2.08	-	133	13.31 ± 3.10	-	133	7.09 ± 2.27	-
*GSTM1* (null)	188	82.19 ± 145.19	1.23E-01	189	4.99 ± 1.95	9.31E-01	194	13.19 ± 3.53	7.38E-01	194	6.94 ± 2.24	5.56E-01
*GSTT1* (plus)	182	91.47 ± 137.03	-	181	5.03 ± 2.13	-	190	13.08 ± 3.47	-	190	6.81 ± 2.10	-
*GSTT1* (null)	133	94.57 ± 160.93	8.58E-01	134	4.95 ± 1.81	7.13E-01	137	13.45 ± 3.19	3.20E-01	137	7.26 ± 2.43	8.41E-02
EPHX (Low)	197	84.89 ± 127.59	-	198	4.99 ± 2.00	-	207	13.40 ± 3.53	-	207	7.05 ± 2.32	-
EPHX (Medium)	91	100.63 ± 166.33	4.25E-01	90	5.14 ± 2.05	5.57E-01	93	12.91 ± 3.25	2.45E-01	93	6.94 ± 2.13	6.94E-01
EPHX (High)	27	123.89 ± 205.08	3.44E-01	27	4.50 ± 1.79	2.01E-01	27	13.11 ± 2.17	5.53E-01	27	6.81 ± 2.18	5.96E-01
EPHX (Medium+High)	118	105.95 ± 175.28	2.57E-01	117	5.00 ± 2.01	9.83E-01	120	12.96 ± 3.03	2.33E-01	120	6.91 ± 2.14	5.86E-01
*CYP2E1* Rsal (c1/c1)	204	101.78 ± 167.21	-	204	5.01 ± 2.09	-	212	13.24 ± 3.33	-	212	6.93 ± 2.31	-
*CYP2E1* Rsal (c1/c2)	101	80.45 ± 103.15	1.72E-01	101	5.01 ± 1.83	9.93E-01	105	13.13 ± 3.37	7.84E-01	105	7.19 ± 2.12	3.21E-01
*CYP2E1* Rsal (c2/c2)	10	33.67 ± 36.10	1.85E-04***	10	4.44 ± 1.76	3.44E-01	10	14.24 ± 4.00	4.56E-01	10	6.52 ± 2.34	6.01E-01
*CYP2E1* Rsal (c1/c2+c2/c2)	111	76.24 ± 99.80	9.09E-02	111	4.96 ± 1.82	8.14E-01	115	13.23 ± 3.42	9.73E-01	115	7.13 ± 2.14	4.30E-01
*CYP2E1* 96bp Insert (0)	195	86.45 ± 135.40	-	195	4.99 ± 1.85	-	204	13.26 ± 3.35	-	204	7.09 ± 2.28	-
*CYP2E1* 96bp Insert (1)	104	89.74 ± 126.10	8.34E-01	104	5.02 ± 2.31	9.16E-01	107	12.92 ± 3.18	3.83E-01	107	6.95 ± 2.23	5.93E-01
*CYP2E1* 96bp Insert (2)	16	189.56 ± 312.50	2.09E-01	16	4.81 ± 1.54	6.52E-01	16	15.09 ± 4.17	1.06E-01	16	6.25 ± 1.96	1.19E-01
*CYP2E1* 96bp Insert (1+2)	120	103.05 ± 165.03	3.55E-01	120	4.99 ± 2.22	9.97E-01	123	13.20 ± 3.39	8.83E-01	123	6.85 ± 2.21	3.58E-01
*CYP2E1* Dral (D/D)	189	98.53 ± 157.04	-	189	4.97 ± 2.04	-	195	13.27 ± 3.34	-	195	6.95 ± 2.33	-
*CYP2E1* Dral (D/C)	108	91.40 ± 140.22	6.87E-01	108	5.01 ± 1.93	8.42E-01	114	12.99 ± 3.24	4.61E-01	114	7.09 ± 2.10	5.89E-01
*CYP2E1* Dral (C/C)	18	40.65 ± 36.31	9.80E-05***	18	5.18 ± 2.04	6.81E-01	18	14.47 ± 4.13	2.46E-01	18	7.04 ± 2.45	8.81E-01
*CYP2E1* Dral (D/C+C/C)	126	84.15 ± 131.63	3.80E-01	126	5.04 ± 1.94	7.56E-01	132	13.19 ± 3.40	8.28E-01	132	7.08 ± 2.14	5.96E-01

*** *p* < 0.001, based on independent samples *t* - test, and compared with the corresponding wild-type genotype.

The above results suggested a prominent role of *ALDH2* polymorphisms, which might overshadow the effects of other genetic polymorphisms on styrene metabolism and styrene-related DNA damage. So we further stratified the exposed samples by focusing on the wild-type genotype ALDH2 *1/*1 and re-analyzed the effects of other genetic polymorphisms (Table 6). For example, *CYP2E1* Rsal variant homozygous c2/c2 and heterozygous c1/c2 genotypes exhibited significantly lower mean level of urinary MA+PGA than wild-type c1/ c2 genotypes. Also, *GSTM1* null individuals had lower MA+PGA concentration than *GSTM1* plus ones. When compared with respective wild-type genotypes, *CYP2E1* Rsal and 96bp insert variant homozygous genotypes were found to be related with decreased DNA-base oxidation in leukocytes.

**Table 6:** Analysis of the influence of *GSTM1, GSTT1, EPHX1* and *CYP2E1* polymorphisms on urinary excretion of styrene specific metabolites and the levels of various parameters of genetic damage in exposed workers carrying ALDH2 *1/*1

styrene-exposed workers carrying ALDH2*1/*1	Urinary MA + PGA (mg/g creatinine)			Urinary 8-OH-dG (ng/mg creatinine)			Tail Intensity			Net Fpg DNA damage		
	No.	Mean ± SD	*P*-value	No.	Mean ± SD	*P*-value	No.	Mean ± SD	*P*-value	No.	Mean ± SD	*P*-value
GSTM1 (plus)	85	136.07 ± 173.15	-	85	4.68 ± 1.74	-	88	12.78 ± 2.89	-	88	6.82 ± 2.29	-
GSTM1 (null)	136	88.31 ± 158.13	4.08E-02*	136	4.79 ± 1.74	6.57E-01	141	12.54 ± 3.42	5.70E-01	141	6.65 ± 2.17	5.77E-01
GSTT1 (plus)	141	95.46 ± 143.17	-	141	4.80 ± 1.81	-	148	12.73 ± 3.38	-	148	6.68 ± 2.07	-
GSTT1 (null)	80	126.44 ± 197.93	2.21E-01	80	4.64 ± 1.61	4.94E-01	81	12.45 ± 2.92	5.09E-01	81	6.78 ± 2.47	7.66E-01
EPHX1 (Low)	136	94.90 ± 138.28	-	136	4.65 ± 1.54	-	142	12.82 ± 3.43	-	142	6.83 ± 2.32	-
EPHX1 (Medium)	66	116.41 ± 190.14	4.14E-01	66	5.03 ± 2.09	2.01E-01	68	12.21 ± 3.08	1.96E-01	68	6.61 ± 2.06	4.81E-01
EPHX1 (High)	19	157.20 ± 236.70	2.76E-01	19	4.43 ± 1.76	6.00E-01	19	12.65 ± 1.79	7.26E-01	19	6.28 ± 1.97	2.75E-01
EPHX1 (Medium+High)	85	125.52 ± 200.68	2.19E-01	85	4.89 ± 2.02	3.54E-01	87	12.31 ± 2.84	2.19E-01	87	6.54 ± 2.03	3.14E-01
CYP2E1 Rsal (c1/c1)	146	122.31 ± 192.00	-	146	4.77 ± 1.70	-	151	12.66 ± 3.10	-	151	6.62 ± 2.28	-
CYP2E1 Rsal (c1/c2)	68	79.94 ± 89.98	2.90E-02*	68	4.77 ± 1.88	9.98E-01	71	12.45 ± 3.34	6.60E-01	71	7.07 ± 2.09	1.53E-01
CYP2E1 Rsal (c2/c2)	7	40.42 ± 42.08	1.36E-03**	7	4.07 ± 0.96	1.09E-01	7	13.76 ± 4.73	5.64E-01	7	5.27 ± 1.19	2.31E-02*
CYP2E1 Rsal (c1/c2+c2/c2)	75	76.25 ± 87.22	1.52E-02*	75	4.70 ± 1.82	7.99E-01	78	12.57 ± 3.47	8.47E-01	78	6.91 ± 2.09	3.46E-01
CYP2E1 96bp Insert (0)	139	94.25 ± 146.42	-	139	4.84 ± 1.83	-	145	12.53 ± 3.23	-	145	6.84 ± 2.27	-
CYP2E1 96bp Insert (1)	71	107.48 ± 145.68	5.35E-01	71	4.64 ± 1.65	4.08E-01	73	12.61 ± 3.06	8.54E-01	73	6.67 ± 2.12	5.88E-01
CYP2E1 96bp Insert (2)	11	258.55 ± 359.73	1.62E-01	11	4.24 ± 0.82	5.14E-02	11	14.09 ± 4.05	2.39E-01	11	5.52 ± 1.81	4.12E-02*
CYP2E1 96bp Insert (1+2)	82	127.75 ± 192.36	1.76E-01	82	4.58 ± 1.57	2.63E-01	84	12.80 ± 3.22	5.33E-01	84	6.52 ± 2.11	2.85E-01
CYP2E1 Dral (D/D)	139	115.20 ± 177.91	-	139	4.72 ± 1.68	-	143	12.72 ± 3.08	-	143	6.69 ± 2.30	-
CYP2E1 Dral (D/C)	71	99.81 ± 149.81	5.10E-01	71	4.88 ± 1.92	5.33E-01	75	12.44 ± 3.36	5.61E-01	75	6.89 ± 2.09	5.15E-01
CYP2E1 Dral (C/C)	11	43.19 ± 40.84	5.13E-04***	11	4.25 ± 1.13	2.26E-01	11	12.74 ± 4.29	9.86E-01	11	5.89 ± 1.79	1.88E-01
CYP2E1 Dral (D/C+C/C)	82	92.22 ± 141.34	2.91E-01	82	4.80 ± 1.84	7.39E-01	86	12.48 ± 3.47	6.07E-01	86	6.76 ± 2.07	8.03E-01

* *p* < 0.05,

** *p* < 0.01,

*** *p* < 0.001, based on independent samples *t* - test, and compared with the corresponding wild-type genotype.

## DISCUSSION

In this study, we investigated the urinary excretion of styrene metabolites and various parameters of DNA damage, and analyzed if the genetic polymorphisms of styrene-metabolizing enzymes exert any modification. Considering the confusing results about the role of genetic polymorphisms as reported, we felt that a large number of styrene-exposed workers and comparable controls should be necessary to improve the statistical power. To our best knowledge, this is the largest sample size so far used for studying the functional relevance of these polymorphisms in the occupational exposure of styrene.

One study [21] proposed that styrene exposure may increase oxidative stress and in turn result in the oxidative DNA damage. Using Fpg-modified comet assay, we found that DNA-base oxidation in leukocytes was induced by styrene exposure. The result was consistent with prior studies [22, 23], which demonstrated that occupational exposure to styrene in workers significantly increased the levels of leukocyte 8-OH-dG.

Instead of 8-OH-dG in leukocytes, we analyzed urinary 8-OH-dG to evaluate the oxidative stress of whole body. The results were in line with other two biomarkers (DNA strand breaks and DNA-base oxidation). Similar results were also observed in an earlier study [24], though not reaching statistical significance with controls. This discrepancy could be explained by variations in sample size, exposure levels of styrene, ethnicity, and measurement methods of urinary 8-OH-dG. Taken together, our data and previous observations suggested that oxidative stress played a key role in styrene-induced genetic damage.

Consistent with many previous studies [9], our current results indicated a positive correlation between styrene exposure and DNA strand breaks in leukocytes. However, ethnic differences in styrene-related health outcomes may exist. For instance, one study showed that high exposure styrene (46.74 ppm) in Caucasians induced significant DNA damage and decreased the DNA repair capacities [25], but not in Caucasians exposed to lower levels of styrene (19.13 ppm) [26]. A recent study, however, showed that exposure to styrene of less than 20 ppm was associated with significantly increased genotoxic risk and decreased DNA repair capacity in Asian workers [22]. These inconsistent results suggest that East Asians are more sensitive to styrene-induced toxicity.

Ethnic differences were also found in metabolism of styrene, as a previous study described lower concentrations of urinary PGA in Orientals than in Caucasians [20]. Such difference may be explained by physiological factors such as body size, body composition, and rental function, as well as genetic background.

In this study, we focused on the effects of *ALDH2* polymorphisms on styrene metabolism and styrene-related DNA damage. Our results indicated that variant allele ALDH2 *2 could significantly decrease the excretion of urinary MA+PGA in workers exposed to styrene. It is believed that the *ALDH2* polymorphisms alter the enzyme activity and influence the oxidation of styrene glycol to MA, which may subsequently result in the accumulation of upstream product of MA, i.e., styrene glycoaldehydes. In animal experiments of styrene exposure by either oral administration or inhalation, our results also showed that *Aldh2* knockout mice had lower concentrations of MA and PGA and higher DNA damage than those of wild-type mice (unpublished data). Also, previous studies [18, 27–29] have demonstrated that clearance of other aldehyde intermediates (e.g. acetaldehyde, ethylene glycoaldehyde, methoxyacetaldehyde, benzaldehyde, and propionaldehyde) were modulated by *ALDH2* polymorphisms. These strong evidences together showed that individuals with *ALDH2* variant allele have more difficulty of clearing aldehydes. Furthermore, accumulation of the aldehyde intermediate appeared to be related with increased DNA damage induced by styrene in occupational workers, as allelic variation of *ALDH2* increased the levels of DNA strand breaks, DNA-base oxidation of leukocytes and urinary 8-OH-dG as comparison to wild-type genotype. It is well known that aldehydes are highly reactive molecules that react with biomacromolecules (e.g. proteins, DNA) to yield covalent adducts, which are likely to cause a series of functional alteration of pathology [30, 31]. Existing evidences showed that aldehydes (such as formaldehyde, acetaldehyde, crotonaldehyde, furfural, acrolein, methoxyacetaldehyde, benzaldehyde and aldehydes intermediates of some drugs) could cause toxic side-effect and even increased cancer risk. The metabolism and toxic effects of some aldehydes have been proven to be modified by *ALDH2* polymorphisms [12, 32–37]. So the regulatory role of *ALDH2* polymorphisms in detoxification of aldehyde intermediate would be crucial in understanding the mechanism underlying the inter-individual difference on styrene-induced toxicity. In addition, increased genetic toxicity of styrene in relation to *ALDH2* polymorphisms is possibly related to other toxic products of styrene, such as styrene-7,8-oxide (SO), which is well documented for playing an important role in risk assessment of styrene. Since we cannot exclude the possibility that ALDH2 activity might also influence the metabolic fate of upstream products of aldehyde intermediate, more experiments with animal or *in vitro* system are needed to address this concern.

Regarding other polymorphisms of enzymes, our results suggested that ALDH2 activity appeared to mask some modifications of *CYP2E1* polymorphisms on styrene metabolism and genotoxic effects, since we found that heterozygous c1/c2 of *CYP2E1* significantly decreased the excretion of urinary MA+PGA among the styrene-exposed workers bearing ALDH2 *1/*1 genotype. Moreover, the similar results were also found in styrene-related DNA damage. Our positive results regarding association between *CYP2E1* polymorphisms and styrene metabolism can be explained by an earlier study, showing that *CYP2E1* c1/c2 decreased the expression of CYP2E1 mRNA [38].

GSTs could protect from styrene-induced toxicity through its catalyzing GSH conjugation of SO. We found that *GSTM1* null genotype significantly decreased the MA+PGA concentrations in individuals carrying ALDH2*1/*1, but not in all exposed workers. Also, homozygous deletions of *GSTM1* and *GSTT1* did not significantly affect the genetic damage induced by styrene exposure. Previous studies indicated the contradictory results about associations between *GSTs* polymorphisms and biomarkers for styrene metabolism and genotoxic effects [9]. In fact, these limited data are difficult to be explained using effects of *GSTs* polymorphisms on styrene metabolism alone, because GSH conjugation of SO via GSTs accounts for only about 1% of all absorbed styrene [1].

In theory, the *EPHX1* gene encoding microsomal epoxide hydrolase is considered as a more important enzyme for detoxifying styrene-induced toxicity than GSTs, since EPHX is a key enzyme involved in the metabolism of SO. A review [9], however, suggested that no solid evidences showed that the activity of EPHX, based on the *EPHX1* polymorphisms in exon 3 and exon 4, influenced the levels of urinary MA+PGA and was related to risk of SO toxicity in styrene-exposed workers. Our data indicated the correlation between higher activity of EPHX and increased urinary MA+PGA concentrations and decreased genetic damage, even though statistical significance was not achieved.

In general, our study initially identified the *in vivo* associations between *ALDH2* polymorphisms and biomarkers of styrene metabolisms and related genotoxic effects in exposed workers. In the meantime, some results need to be further confirmed. First of all, because of the instability of aldehyde and some technical difficulties, we used urinary MA+PGA as the indirect biomarker reflecting the accumulation of styrene glycoaldehyde, rather than directly measuring the aldehyde in blood. Secondly, additional *in vitro* and *in vivo* studies are still needed to investigate the toxicological mechanisms related to styrene glycoaldehyde. Furthermore, a well-designed animal model such as *Aldh2* knockout mouse is required to confirm *ALDH2* polymorphisms involved in inter-individual variation in the study of styrene exposure, in order to minimize potential confounding factors that can be hardly avoided in human epidemiological studies. Despite these, the results obtained in this study clearly suggested that *ALDH2* polymorphisms play a prominent role in styrene metabolism and related genotoxicity. Combined with accumulated evidences regarding the function of *ALDH2* in the detoxification of toxic and carcinogenic aldehyde intermediates [12, 39, 40], it is reasonable to consider individual *ALDH2* polymorphisms in quantitatively assessing the risk of styrene exposure.

## MATERIALS AND METHODS

### Study subjects

This cross-sectional study was approved by the Research Ethics Committees of the National Institute of Occupational Safety and Health, Japan, and National Institute for Occupational Health and Poison Control, Chinese Center for Disease Control and Prevention. In this study, all the participants were interviewed by an occupational physician using a detailed questionnaire, which included demographic information, occupational history, individual lifestyle, and personal medical history, and styrene-exposed participants worked for at least six months. The study was conducted on healthy individuals and exclusion criteria included recent exposure to mutagenic agents (such as X-ray), current drug use, and recent viral infections. Finally, 329 styrene-exposed workers were recruited from three different workplaces, defined as workplace A, B and C, of the fiberglass-reinforced plastic factory in China, and 152 unexposed clerks were also recruited from the same factory.

### Blood and urine sampling

In an annual physical examination, blood and urine samples were collected from 8 a.m to 11 a.m on Wednesday, Thursday and Friday, before subjects started their work. All samples were placed in an icebox and were immediately transported to the National Institute of Occupational Health and Poison Control, China CDC. Part of the blood samples was immediately used for the analysis of comet assay on the same day of sampling. The remaining blood, serum and urine samples were stored at – 80°C. All samples were coded and analyzed under blind conditions.

### Styrene exposure at the workplace

In each workplace, 10 workers located at different sites were randomly selected to wear personal dosimeters (3M, St. Paul, MN) close to the breathing zone for 8 h (an entire work shift). The concentration of airborne styrene at the workplace was analyzed by an Agilent gas chromatography (Santa Clara, CA) with the column HP-INNOWax according to the National Institute for Occupational Safety and Health method 1501 [41].

### Analysis of urinary styrene metabolites

Urinary MA and PGA concentrations were determined as described in a prior study [42]. An aliquot of 100 μl urine sample was diluted with 1ml pure water. Mixed solution was filtered using syringe-driven filter unit (Millipore, Billerica, MA). 20 μl of each sample was injected into the HPLC system (Agilent model 1100 series) equipped with a UV detector. The column (GL Sciences, Tokyo, Japan) used was 150 mm in length and 4.6 mm in inner diameter, and was packed with inertsil ODS (diameter of the granules, 5 μm). The final concentrations of MA and PGA were expressed as mg/g Creatinine (Cr.), and Cr. concentrations in the urine samples were determined according to Jaffe's method. Sample with Cr. Concentrations lower than 0.3 g/L or higher than 3.0 g/L were excluded from statistical analysis according to the American Conference of Governmental Industrial Hygienists recommendation [43].

### Measurement of urinary 8-OH-dG

Urinary 8-OH-dG concentration was determined by HPLC-ECD using a two-separation method as described previously [44] and expressed as mg/g Creatinine. Urine samples were thawed and mix with a solution containing the ribonucleoside marker 8-hydroxygunosine (8- OH- G). After mixed solutions were put at 4°C for 2 h, then centrifuged at 13,000 rpm for 5 min, and the 20 μl aliquots of the supernatants were used for the analysis. Firstly, 8-OH-dG fraction in supernatant was separated by an anion exchange column (MCI GEL CA08F, 7 μm, 1.5 × 150 mm), and collected based on the 8-OH-G peak. Secondly, collected 8-OH-dG fraction was further separated by a reverse phase column (Shiseido, Capcell Pak C18, 5 μm, 4.6 × 250 mm), then and was detected by a Coulochem II electrochemical detector (ESA) with a guard cell (5020) and an analytical cell (5011). Applied voltage was set at 400 mV in guard cell and was set at 190 mV and 350 mV in analytical cell. The value of urinary 8-OH-dG was expressed as mg/g Cr.

### Standard comet assay

The comet assay was carried out under alkaline conditions, basically as a prior description [45] with some modification [46]. Finally, 100 cells were scored per sample using the Comet Assay IV capture system (Perceptive Instruments, Suffolk, UK). The tail intensity (TI), defined as the percentage of DNA migrated from the head of the comet into the tail, was measured for each nucleus.

### FPG-modified comet assay

Details of analytical conditions about the formamido-pyrimidine-DNA-glycosylase (FPG)-modified comet assay were described in a previous study [47]. Briefly, samples were treated with 1 U FPG enzyme (Trevigen, Gaithersburg, MD, USA) per gel in 50 μl FPG buffer (40 mmol/L HEPES, 0.1 mol/L KCl, 0.5 mmol/L EDTA, 0.2 mg/mL bovine serum albumin, pH 8) for 60 min at 37°C, the following procedure was in line with the standard come assay. The net FPG DNA damage was considered as the differences in measured TI values between samples obtained with standard alkaline comet assay (basic DNA damage) and FPG-modified comet assay (total DNA damage).

### DNA isolation and genotyping

Genomic DNA was isolated from whole blood samples using EZ1 Blood DNA kit (Qiagen, Hilden, Germany). Genotyping for *ALDH2*, *CYP2E1* (5′-flanking region, RsaI/PstI, 96-bp insertion, and intron 6, DraI), *EPHX* (exon 3, Tyr113His, EcoRV and exon 4, His139Arg, RsaI), *GSTM1,* and *GSTT1* was carried out as described previously [48–52]. Approximately 30% of all samples with clear genotypes were regenotyped once in independent experiments, and repeat results were 100% consistent with previous results. To Inconclusive samples were reanalyzed twice or more times, and only concordant results from the analyses were accepted.

The EPHX enzymatic activity in individuals was classified based on the results of genotyping of polymorphisms in exons 3 and 4 [9]. Low activity: His/His–His/His; His/His–His/Arg; Tyr/His–His/His; His/His–Arg/Arg. Medium activity: Tyr/Tyr–His/His; Tyr/His–His/Arg; Tyr/His–Arg/Arg. High activity: Tyr/Tyr–Arg/Arg; Tyr/Tyr–His/Arg.

### Statistical analysis

The deviation of genotype distribution from Hardy-Weinberg equilibrium was examined with Chi-square test in both control and exposed groups. Then, the independent *t* - test was performed to compare the levels of biomarkers (i.e. DNA strand breaks, DNA-base oxidation in leukocytes, urinary 8-OH-dG concentrations, and urinary MA+PGA) (1) between exposure and control groups, and (2) between the subjects carrying mutant allele and those of wild-type genotype. The Levene's test was used to assess the equality of variances of data. If necessary, the data were transformed in order to restore equal variances. A *p*-value < 0.05 was used as the threshold of statistical significance. In addition, the styrene-*ALDH2* genotypes interaction on the degree of genetic damage was further confirmed by multivariate linear regression models adjusting the effects of sex, age, years of employment, smoking and alcohol drinking. All analyses were performed using the epicalc package of R software (cran.r-project.org/package=epical).

## SUPPLEMENTARY FIGURES AND TABLES


