# Supplementary material for: Significant association between decreased ALDH2 activity and increased sensitivity to genotoxic effects in workers occupationally exposed to styrene
Source: Oncotarget. 2016 May 20;7(25):38224–34. doi: 10.18632/oncotarget.9502 (PMC5122384; doi:10.18632/oncotarget.9502)
Supplement: Supplementary file 1 [file oncotarget-07-38224-s001.pdf]

Significant association between decreased ALDH2 activity and increased sensitivity to genotoxic effects in workers occupationally exposed to styrene

Supplementary Materials

Supplementary Table S1: Distribution of styrene-metabolizing genes polymorphisms in this study population

Genotypes	Study subjects														
	Controls			Styrene-exposed workers											
				Workplace A			Workplace B			Workplace C			All exposed		
	No.	Frequency %	pHWE	No.	Frequency %	pHWE	No.	Frequency %	pHWE	No.	Frequency %	pHWE	No.	Frequency %	pHWE
ALDH2 1/1**	96	66.21		83	69.75		83	72.81		63	67.02		229	70.03	
ALDH2 1/2**	43	29.66		31	26.05		29	25.44		29	30.85		89	27.22	
ALDH2 2/2**	6	4.14	9.32E-01	5	4.20	8.82E-01	2	1.75	9.93E-01	2	2.13	8.70E-01	9	2.75	1.00E+00
GSTM1 (plus)	58	40.00		46	38.66		48	42.11		39	41.49		133	40.67	
GSTM1 (null)	87	60.00	–	73	61.34	–	66	57.89	–	55	58.51	–	194	59.33	–
GSTT1 (plus)	83	57.24		69	57.98		61	53.51		60	63.83		190	58.10	
GSTT1 (null)	62	42.76	–	50	42.02	–	53	46.49	–	34	36.17	–	137	41.90	–
EPHX1 Exon 3 (Tyr/Tyr)	44	30.34		32	26.89		40	35.09		32	34.04		104	31.80	
EPHX1 Exon 3 (Tyr/His)	47	32.41		37	31.09		36	31.58		28	29.79		101	30.89	
EPHX1 Exon 3 (His/His)	54	37.24	9.65E-03**	50	42.02	1.59E-02*	38	33.33	2.15E-02*	34	36.17	2.18E-02*	122	37.31	4.78E-06***
EPHX1 Exon 4 (His/His)	120	82.76		94	78.99		92	80.70		77	81.91		263	80.43	
EPHX1 Exon 4 (His/Arg)	23	15.86		20	16.81		19	16.67		17	18.09		56	17.13	
EPHX1 Exon 4 (Arg/Arg)	2	1.38	8.37E-01	5	4.20	3.47E-01	3	2.63	5.39E-01	0	0.00	5.68E-01	8	2.45	3.87E-01
CYP2E1 RsaI (c1/c1)	82	56.55		78	65.55		74	64.91		60	63.83		212	64.83	
CYP2E1 RsaI (c1/c2)	51	35.17		40	33.61		34	29.82		31	32.98		105	32.11	
CYP2E1 RsaI (c2/c2)	12	8.28	8.04E-01	1	0.84	3.35E-01	6	5.26	8.96E-01	3	3.19	9.22E-01	10	3.06	8.74E-01
CYP2E1 96bp Insert (0)	92	63.45		76	63.87		73	64.04		55	58.51		204	62.39	
CYP2E1 96bp Insert (1)	49	33.79		37	31.09		33	28.95		37	39.36		107	32.72	
CYP2E1 96bp Insert (2)	4	2.76	7.74E-01	6	5.04	9.28E-01	8	7.02	5.76E-01	2	2.13	5.89E-01	16	4.89	9.74E-01
CYP2E1 DraI (D/D)	78	53.79		74	62.18		67	58.77		54	57.45		195	59.63	
CYP2E1 DraI (D/C)	56	38.62		40	33.61		38	33.33		36	38.30		114	34.86	
CYP2E1 DraI (C/C)	11	7.59	9.71E-01	5	4.20	9.96E-01	9	7.89	7.87E-01	4	4.26	9.15E-01	18	5.50	9.76E-01

pHWE: *p*-Value of the Hardy-Weinberg Equilibrium (*$p < 0.05$, **$p < 0.01$, ***$p < 0.001$). All SNPs except *EPHX1* exon 3 showed that their allelic frequencies were in accordance with the Hardy-Weinberg equilibrium (HWE) in controls, exposed groups, and in subgroups. However, the inequilibrious distribution of *EPHX1* exon 3 genotypes should not affect the reliability of the statistical results, since our interest was the difference of biomarker levels between different genotypes within the same group, rather the difference of genotype distribution between individual groups.

Supplementary Table S2: Effects of *GSTM1, GSTT1, EPHX1* and *CYP2E1* polymorphisms on the levels of various parameters of genetic damage in controls

Controls	Urinary 8-OH-dG (ng/mg creatinine)			Tail Intensity			Net Fpg DNA damage		
	No.	Mean ± SD	*P*-value	No.	Mean ± SD	*P*-value	No.	Mean ± SD	*P*-value
GSTM1 (plus)	57	4.06 ± 1.61	-	58	11.92 ± 3.76	-	58	6.02 ± 1.85	-
GSTM1 (null)	84	3.76 ± 1.24	2.48E-01	87	11.56 ± 3.98	5.84E-01	87	6.12 ± 1.98	7.58E-01
GSTT1 (plus)	81	3.78 ± 1.47	-	83	11.69 ± 4.15	-	83	6.06 ± 1.94	-
GSTT1 (null)	60	4.02 ± 1.31	3.25E-01	62	11.73 ± 3.53	9.46E-01	62	6.11 ± 1.91	8.70E-01
EPHX (Low)	90	3.90 ± 1.21	-	94	11.76 ± 3.90	-	94	6.00 ± 1.98	-
EPHX (Medium)	39	3.96 ± 1.90	8.56E-01	39	12.02 ± 3.87	7.22E-01	39	6.43 ± 1.67	2.06E-01
EPHX (High)	12	3.50 ± 0.86	1.72E-01	12	10.28 ± 3.84	2.30E-01	12	5.56 ± 2.19	5.19E-01
EPHX (Medium+High)	51	3.85 ± 1.71	8.62E-01	51	11.61 ± 3.90	8.30E-01	51	6.23 ± 1.82	4.94E-01
CYP2E1 Rsal (c1/c1)	78	3.77 ± 1.22	-	82	11.48 ± 3.48	-	82	6.33 ± 1.87	-
CYP2E1 Rsal (c1/c2)	51	3.88 ± 1.48	6.71E-01	51	11.76 ± 4.38	6.98E-01	51	5.63 ± 1.99	4.49E-02*
CYP2E1 Rsal (c2/c2)	12	4.60 ± 2.03	1.92E-01	12	13.00 ± 4.39	2.72E-01	12	6.30 ± 1.76	9.50E-01
CYP2E1 Rsal (c1/c2+c2/c2)	63	4.02 ± 1.60	3.21E-01	63	12.00 ± 4.37	4.43E-01	63	5.75 ± 1.96	7.48E-02
CYP2E1 96 bp Insert (0)	89	3.77 ± 1.16	-	92	11.84 ± 3.93	-	92	5.94 ± 1.95	-
CYP2E1 96 bp Insert (1)	48	3.89 ± 1.53	6.16E-01	49	11.30 ± 3.78	4.30E-01	49	6.28 ± 1.92	3.32E-01
CYP2E1 96 bp Insert (2)	4	6.30 ± 2.75	1.62E-01	4	13.73 ± 4.18	4.36E-01	4	6.81 ± 1.00	1.81E-01
CYP2E1 96 bp Insert (1+2)	52	4.08 ± 1.74	2.52E-01	53	11.48 ± 3.83	5.96E-01	53	6.32 ± 1.86	2.56E-01
CYP2E1 Dral (D/D)	75	3.79 ± 1.43	-	78	11.37 ± 3.66	-	78	6.14 ± 1.73	-
CYP2E1 Dral (D/C)	55	3.89 ± 1.45	6.81E-01	56	11.75 ± 3.85	5.69E-01	56	6.02 ± 2.20	7.46E-01
CYP2E1 Dral (C/C)	11	4.49 ± 0.83	2.86E-02*	11	13.84 ± 5.19	1.54E-01	11	5.94 ± 1.85	7.44E-01
CYP2E1 Dral (D/C+C/C)	66	3.99 ± 1.38	3.86E-01	67	12.10 ± 4.13	2.71E-01	67	6.01 ± 2.14	6.95E-01

*$p < 0.05$, based on independent samples *t* - test, and compared with the corresponding wild-type genotype.

Supplementary Table S3: Effects of *GSTM1, GSTT1, EPHX1* and *CYP2E1* polymorphisms on urinary excretion of styrene specific metabolites and the levels of various parameters of genetic damage in styrene-exposed workers at workplace A

styrene-exposed workers	Urinary MA+PGA (mg/g creatinine)			Urinary 8-OH-dG (ng/mg creatinine)			Tail Intensity			Net Fpg DNA damage		
	No.	Mean ± SD	*P*-value	No.	Mean ± SD	*P*-value	No.	Mean ± SD	*P*-value	No.	Mean ± SD	*P*-value
GSTM1 (plus)	43	51.90 ± 92.12	-	43	4.44 ± 1.42	-	46	12.84 ± 3.53	-	46	6.75 ± 2.21	-
GSTM1 (null)	69	86.11 ± 181.98	1.92E-01	70	4.53 ± 1.73	7.68E-01	73	12.86 ± 3.91	9.79E-01	73	6.68 ± 2.18	8.50E-01
GSTT1 (plus)	63	68.40 ± 123.12	-	63	4.37 ± 1.61	-	69	12.97 ± 4.15	-	69	6.48 ± 2.03	-
GSTT1 (null)	49	78.86 ± 188.00	7.37E-01	50	4.65 ± 1.62	3.74E-01	50	12.69 ± 3.15	6.73E-01	50	7.02 ± 2.36	1.90E-01
EPHX (Low)	76	66.50 ± 117.31	-	77	4.45 ± 1.54	-	82	12.93 ± 3.98	-	82	6.76 ± 2.20	-
EPHX (Medium)	27	100.26 ± 245.78	4.98E-01	27	4.71 ± 2.00	5.44E-01	28	12.24 ± 3.32	3.71E-01	28	6.38 ± 2.04	4.12E-01
EPHX (High)	9	45.83 ± 28.82	2.17E-01	9	4.27 ± 0.96	6.37E-01	9	13.99 ± 2.75	3.20E-01	9	7.24 ± 2.58	6.01E-01
EPHX (Medium+High)	36	86.65 ± 213.63	5.99E-01	36	4.6 ± 1.79	6.67E-01	37	12.67 ± 3.24	7.01E-01	37	6.59 ± 2.17	7.01E-01
CYP2E1 Rsal (c1/c1)	73	84.53 ± 186.09	-	74	4.44 ± 1.66	-	78	12.85 ± 3.83	-	78	6.66 ± 2.28	-
CYP2E1 Rsal (c1/c2)	38	52.18 ± 56.24	1.74E-01	38	4.66 ± 1.51	4.77E-01	40	12.83 ± 3.70	9.82E-01	40	6.75 ± 2.02	8.26E-01
CYP2E1 Rsal (c2/c2)	1	20.16 ± NA	-	1	2.03 ± NA	-	1	13.64 ± NA	-	1	8.61 ± NA	-
CYP2E1 Rsal (c1/c2+c2/c2)	39	51.36 ± 55.73	1.62E-01	39	4.6 ± 1.55	6.23E-01	41	12.85 ± 3.65	9.96E-01	41	6.79 ± 2.01	7.39E-01
CYP2E1 96 bp Insert (0)	71	65.65 ± 159.04	-	72	4.57 ± 1.66	-	76	12.76 ± 3.73	-	76	6.78 ± 2.18	-
CYP2E1 96 bp Insert (1)	35	92.93 ± 156.08	4.03E-01	35	4.29 ± 1.65	4.14E-01	37	12.92 ± 3.76	8.34E-01	37	6.65 ± 2.31	7.77E-01
CYP2E1 96 bp Insert (2)	6	43.25 ± 59.01	4.77E-01	6	4.82 ± 0.55	4.18E-01	6	13.55 ± 4.54	6.95E-01	6	6.10 ± 1.47	3.29E-01

CYP2E1 96 bp Insert (1+2)	41	85.66 ± 146.48	5.02E-01	41	4.37 ± 1.54	5.16E-01	43	13.01 ± 3.82	7.35E-01	43	6.57 ± 2.21	6.24E-01
CYP2E1 Dral (D/D)	70	86.90 ± 189.77	-	71	4.44 ± 1.70	-	74	12.88 ± 3.85	-	74	6.70 ± 2.32	-
CYP2E1 Dral (D/C)	37	49.69 ± 56.27	1.32E-01	37	4.57 ± 1.46	6.86E-01	40	12.77 ± 3.69	8.81E-01	40	6.53 ± 1.94	6.77E-01
CYP2E1 Dral (C/C)	5	50.48 ± 33.41	1.89E-01	5	4.77 ± 1.79	7.09E-01	5	12.98 ± 3.58	9.56E-01	5	8.14 ± 1.67	1.29E-01
CYP2E1 Dral (D/C+C/C)	42	49.78 ± 53.75	1.28E-01	42	4.59 ± 1.48	6.21E-01	45	12.80 ± 3.64	9.02E-01	45	6.71 ± 1.96	9.84E-01

Supplementary Table S4: Effects of *GSTM1, GSTT1, EPHX1* and *CYP2E1* polymorphisms on urinary excretion of styrene specific metabolites and the levels of various parameters of genetic damage in styrene-exposed workers at workplace B

styrene-exposed workers	Urinary MA+PGA (mg/g creatinine)			Urinary 8-OH-dG (ng/mg creatinine)			Tail Intensity			Net Fpg DNA damage		
	No.	Mean ± SD	*P*-value	No.	Mean ± SD	*P*-value	No.	Mean ± SD	*P*-value	No.	Mean ± SD	*P*-value
GSTM1 (plus)	47	137.6 ± 195.67	-	47	4.63 ± 2.29	-	48	12.60 ± 2.56	-	48	7.08 ± 2.44	-
GSTM1 (null)	64	55.89 ± 101.87	1.11E-02*	64	4.76 ± 1.81	7.48E-01	66	13.33 ± 3.55	2.08E-01	66	6.68 ± 2.29	3.81E-01
GSTT1 (plus)	59	95.75 ± 174.10	-	59	4.73 ± 2.33	-	61	12.63 ± 3.07	-	61	6.66 ± 2.02	-
GSTT1 (null)	52	84.51 ± 127.91	6.97E-01	52	4.68 ± 1.62	8.95E-01	53	13.48 ± 3.28	1.58E-01	53	7.07 ± 2.68	3.76E-01
EPHX (Low)	66	78.68 ± 124.75	-	66	4.96 ± 2.25	-	68	13.25 ± 3.41	-	68	6.93 ± 2.41	-
EPHX (Medium)	35	80.86 ± 122.75	9.33E-01	35	4.60 ± 1.68	3.71E-01	36	12.68 ± 3.08	3.88E-01	36	7.02 ± 2.30	8.55E-01
EPHX (High)	10	202.13 ± 322.33	2.60E-01	10	3.45 ± 0.61	4.18E-05***	10	12.69 ± 1.71	4.17E-01	10	5.66 ± 1.96	8.66E-02
EPHX (Medium+High)	45	107.81 ± 188.40	3.66E-01	45	4.34 ± 1.58	9.45E-02	46	12.69 ± 2.82	3.33E-01	46	6.73 ± 2.28	6.43E-01
CYP2E1 Rsal (c1/c1)	72	100.24 ± 165.24	-	72	4.85 ± 2.28	-	74	13.19 ± 3.16	-	74	6.86 ± 2.36	-
CYP2E1 Rsal (c1/c2)	33	81.56 ± 138.94	5.49E-01	33	4.41 ± 1.33	2.22E-01	34	12.36 ± 2.84	1.78E-01	34	7.08 ± 2.38	6.64E-01
CYP2E1 Rsal (c2/c2)	6	22.53 ± 22.44	6.08E-04***	6	4.62 ± 2.01	7.95E-01	6	14.70 ± 4.74	4.77E-01	6	5.42 ± 1.83	1.16E-01
CYP2E1 Rsal (c1/c2+c2/c2)	39	72.48 ± 129.56	3.32E-01	39	4.45 ± 1.43	2.52E-01	40	12.71 ± 3.23	4.46E-01	40	6.83 ± 2.36	9.41E-01
CYP2E1 96 bp Insert (0)	70	83.3 ± 128.48	-	70	4.60 ± 1.63	-	73	12.88 ± 3.13	-	73	6.96 ± 2.45	-
CYP2E1 96 bp Insert (1)	33	71.14 ± 111.67	6.25E-01	33	4.99 ± 2.71	4.50E-01	33	12.76 ± 2.92	8.42E-01	33	6.80± 2.18	7.37E-01
CYP2E1 96 bp Insert (2)	8	233.18 ± 354.36	2.73E-01	8	4.45 ± 1.94	8.39E-01	8	15.40 ± 4.13	1.34E-01	8	6.12 ± 2.25	3.52E-01
CYP2E1 96 bp Insert (1+2)	41	102.76 ± 190.20	5.63E-01	41	4.89 ± 2.57	5.26E-01	41	13.27 ± 3.30	5.40E-01	41	6.66 ± 2.18	5.15E-01
CYP2E1 Dral (D/D)	65	83.81 ± 118.61	-	65	4.91 ± 2.30	-	67	13.31 ± 3.18	-	67	6.86 ± 2.40	-
CYP2E1 Dral (D/C)	37	119.42 ± 211.72	3.51E-01	37	4.35 ± 1.37	1.28E-01	38	12.07 ± 2.51	3.01E-02*	38	7.15 ± 2.31	5.49E-01
CYP2E1 Dral (C/C)	9	19.74 ± 18.62	1.47E-04***	9	4.71 ± 2.07	7.96E-01	9	14.93 ± 4.62	3.35E-01	9	5.52 ± 1.81	6.80E-02
CYP2E1 Dral (D/C+C/C)	46	99.92 ± 193.71	6.18E-01	46	4.42 ± 1.51	1.81E-01	47	12.62 ± 3.17	2.54E-01	47	6.84 ± 2.30	9.54E-01

*$p < 0.05$, ***$p < 0.001$, based on independent samples *t* - test, and compared with the corresponding wild-type genotype.

Supplementary Table S5: Effects of *GSTM1, GSTT1, EPHX1* and *CYP2E1* polymorphisms on urinary excretion of styrene specific metabolites and the levels of various parameters of genetic damage in styrene-exposed workers at workplace C

styrene-exposed workers	Urinary MA+PGA (mg/g creatinine)			Urinary 8-OH-dG (ng/mg creatinine)			Tail Intensity			Net Fpg DNA damage		
	No.	Mean ± SD	*P*-value	No.	Mean ± SD	*P*-value	No.	Mean ± SD	*P*-value	No.	Mean ± SD	*P*-value
GSTM1 (plus)	37	137.16 ± 117.66	-	36	6.17 ± 2.02	-	39	14.74 ± 2.76	-	39	7.50 ± 2.12	-
GSTM1 (null)	55	107.86 ± 132.95	2.70E-01	55	5.82 ± 2.12	4.41E-01	55	13.46 ± 2.94	3.32E-02*	55	7.60 ± 2.17	8.24E-01
GSTT1 (plus)	60	111.47 ± 104.33	-	59	6.02 ± 2.07	-	60	13.68 ± 2.94	-	60	7.35 ± 2.18	-
GSTT1 (null)	32	134.96 ± 162.45	4.63E-01	32	5.84 ± 2.13	6.98E-01	34	14.54 ± 2.84	1.66E-01	34	7.91 ± 2.05	2.16E-01
EPHX (Low)	55	117.74 ± 140.06	-	55	5.79 ± 2.01	-	57	14.25 ± 2.82	-	57	7.61 ± 2.33	-
EPHX (Medium)	29	124.83 ± 115.94	8.05E-01	28	6.25 ± 2.16	3.61E-01	29	13.85 ± 3.29	5.82E-01	29	7.39 ± 1.96	6.37E-01
EPHX (High)	8	113.91 ± 70.61	9.04E-01	8	6.09 ± 2.42	7.49E-01	8	12.65 ± 1.90	6.00E-02	8	7.76 ± 1.40	8.07E-01
EPHX (Medium+High)	37	122.47 ± 106.98	8.55E-01	36	6.21 ± 2.19	3.61E-01	37	13.59 ± 3.06	2.98E-01	37	7.47 ± 1.84	7.37E-01
CYP2E1 Rsal (c1/c1)	59	124.99 ± 143.24	-	58	5.94 ± 2.06	-	60	13.82 ± 2.76	-	60	7.36 ± 2.28	-
CYP2E1 Rsal (c1/c2)	30	115.05 ± 95.22	6.98E-01	30	6.10 ± 2.21	7.39E-01	31	14.37 ± 3.20	4.20E-01	31	7.88 ± 1.82	2.44E-01
CYP2E1 Rsal (c2/c2)	3	60.45 ± 55.36	1.64E-01	3	4.90 ± 0.74	1.10E-01	3	13.54 ± 3.81	9.12E-01	3	8.03 ± 2.63	7.04E-01
CYP2E1 Rsal (c1/c2+c2/c2)	33	110.09 ± 93.07	5.48E-01	33	5.99 ± 2.14	9.08E-01	34	14.29 ± 3.20	4.69E-01	34	7.89 ± 1.85	2.25E-01
CYP2E1 96bp Insert (0)	54	117.89 ± 103.00	-	53	6.09 ± 1.96	-	55	14.45 ± 2.78	-	55	7.69 ± 2.11	-
CYP2E1 96bp Insert (1)	36	103.70 ± 105.80	5.31E-01	36	5.76 ± 2.29	4.87E-01	37	13.07 ± 2.85	2.42E-02*	37	7.37 ± 2.19	4.89E-01
CYP2E1 96bp Insert (2)	2	454.00 ± 533.75	5.37E-01	2	6.20 ± 1.62	9.40E-01	2	18.46 ± 0.18	2.43E-12***	2	7.16 ± 3.13	8.50E-01
CYP2E1 96bp Insert (1+2)	38	122.13 ± 156.75	8.84E-01	38	5.78 ± 2.25	5.05E-01	39	13.34 ± 3.02	7.57E-02	39	7.36 ± 2.19	4.67E-01
CYP2E1 Dral (D/D)	54	131.32 ± 148.55	-	53	5.74 ± 1.91	-	54	13.76 ± 2.70	-	54	7.39 ± 2.23	-
CYP2E1 Dral (D/C)	34	106.3 ± 91.30	3.30E-01	34	6.22 ± 2.34	3.19E-01	36	14.19 ± 3.12	4.99E-01	36	7.64 ± 1.93	5.74E-01
CYP2E1 Dral (C/C)	4	75.39 ± 44.48	9.35E-02	4	6.72 ± 1.97	3.95E-01	4	15.31 ± 4.19	5.14E-01	4	9.08 ± 2.66	2.95E-01
CYP2E1 Dral (D/C+C/C)	38	103.04 ± 87.67	2.56E-01	38	6.27 ± 2.29	2.43E-01	40	14.30 ± 3.19	3.85E-01	40	7.78 ± 2.02	3.73E-01

*$p < 0.05$, ***$p < 0.001$, based on independent samples *t* - test, and compared with the corresponding wild-type genotype.
